# Global Landscape of Infection-Induced Pulmonary Hypertension

**DOI:** 10.3390/idr17020035

**Published:** 2025-04-17

**Authors:** Ghazwan Butrous

**Affiliations:** 1 Cardiopulmonary Sciences, School of Pharmacy, University of Kent, Canterbury CT2 7NZ, UK; g.butrous@kent.ac.uk; 2 Pulmonary Vascular Research Institute, 5 Tanner Street, London SE1 3LE, UK

**Keywords:** infection, pulmonary hypertension, pulmonary vascular diseases, global

## Abstract

**Introduction**: Infectious diseases significantly impact pulmonary vascular disorders, particularly in developing countries where parasitic infections remain prevalent. These infections constitute a substantial yet frequently overlooked contributor to pulmonary hypertension. **Discussion**: This review examines the prevalence of parasitic lung diseases in regions where communicable infections are endemic and highlights their pathophysiological links to pulmonary hypertension. Schistosomiasis and HIV notably increase pulmonary hypertension risk in these areas. While other infectious diseases may also cause pulmonary vascular lesions, most remain insufficiently studied. The review addresses global epidemiological trends, diagnostic challenges, and recent advancements in understanding the multifaceted origins of pulmonary hypertension. **Conclusion**: The association between parasitic infections and pulmonary hypertension is significant, necessitating a high index of suspicion for pulmonary hypertension in patients with a history of parasitic diseases, especially in endemic regions. More research is needed to understand infection-related pulmonary hypertension mechanisms and reduce its global impact.

## 1. Introduction

Pulmonary hypertension is a serious cardiovascular condition characterized by elevated blood pressure in the pulmonary arteries. This puts a significant strain on the right ventricle, which could lead to heart failure and death [1,2]. The medical community has made significant strides in understanding pulmonary hypertension over the past seven decades, greatly enhancing its definition, classification, and epidemiological assessment.

Infectious diseases, especially in developing countries, have the potential to affect the development of pulmonary vascular disorders. Pulmonary hypertension is a risk factor for schistosomiasis, HIV infection, and other infections [3,4]. Paragonimiasis, Ascaris larval, Trichinella, Strongyloides, and Dirofilariasis are just a few of the many parasites that can infect or colonize pulmonary tissues during their life cycles. The host’s immune and inflammatory responses to these infestations can potentially contribute to pulmonary vascular remodeling through mechanisms such as endothelial dysfunction and hypoxia-induced vasoconstriction. A direct causal link between these parasites and pathological alterations in the pulmonary vasculature has not yet been established through observational, clinical studies, or experimental models. Further research is needed to determine whether various parasitic antigens and secondary mediators can synergistically affect the pathogenesis of pulmonary hypertension. This review provides a summary of the most prevalent parasitic lung diseases that have been studied and reported [3,5].

## 2. Evolution of Pulmonary Hypertension Definition and Classification

The definition and classification of pulmonary hypertension have changed substantially since its initial characterization in 1960. Originally, pulmonary hypertension was defined as a mean pulmonary artery pressure of ≥25 mmHg at rest, measured via right heart catheterization. It was broadly categorized into pre-capillary and post-capillary pulmonary hypertension based on pulmonary artery wedge pressure. The Seventh World Symposium on Pulmonary Hypertension, held in Barcelona, Spain, in June 2024, resulted in adopting a revised definition (Table 1). Pulmonary hypertension is a hemodynamic abnormality with a mean pulmonary artery pressure exceeding 20 mm Hg, assessed through right heart catheterization [6,7].

The updated classification system includes pre-capillary, post-capillary, and combined post- and pre-capillary pulmonary hypertension. The pulmonary artery wedge pressure and pulmonary vascular resistance criteria define each category. This evolution reflects decades of progress, with key milestones achieved during World Symposia held in Evian, France (1998); Venice, Italy (2003); Dana Point, USA (2008); and Nice, France (2013 and 2018). These symposiums refined the classification system to better understand pulmonary hypertension’s complex and diverse nature and underlying causes [7,8].

## 3. Epidemiological Challenges and Advancements

The epidemiology of pulmonary hypertension is complex and multifactorial, presenting significant challenges in determining accurate incidence and prevalence. Diagnostic, demographic, and healthcare factors complicate the collection and interpretation of data. Advances in diagnostic tools, such as cardiac catheterization and echocardiography, have improved the detection and understanding of pulmonary hypertension, influencing its epidemiological landscape.

The primary sources of epidemiological data on pulmonary hypertension are registries, databases, and observational studies. Since establishing the first National Institutes of Health (NIH) pulmonary hypertension registry in the United States, over 20 registries have been developed worldwide, encompassing data from more than 10,000 patients. Due to differences in methodologies, diagnostic criteria, and population representations, these registries tend to reflect regional trends instead of global patterns [9,10].

The Global Burden of Diseases, Injuries, and Risk Factors Study (GBD) is a key contributor to understanding the global impact of pulmonary hypertension [11,12,13]. Despite these advances, challenges such as lack of standardization, delayed diagnosis, and underreporting persist in low-income regions.

Several factors complicate the assessment of pulmonary hypertension epidemiology. The lack of standardized diagnostic criteria and protocols hinders direct comparisons across studies. Increasing awareness and therapeutic advancements have led to more frequent diagnoses in high-income regions with better access to healthcare. Delayed diagnosis is still a significant issue in underdeveloped areas, where limited healthcare resources lead to underreporting [14].

Geographic and environmental factors significantly impact the epidemiology of pulmonary hypertension. Living at high altitudes (above 4000 feet) is strongly associated with an increased risk of pulmonary hypertension. Additionally, pollution, socioeconomic disparities, and variations in substance or illicit drug abuse contribute to regional epidemiological differences.

Future research aims to harmonize diagnostic criteria, improve case detection in low-income regions, and explore genetic, environmental, behavioral, and socioeconomic factors that affect pulmonary hypertension susceptibility. Standardizing registry methodologies is crucial to enabling reliable comparisons and trend analyses. Large-scale studies across diverse populations, particularly in underrepresented regions like Asia and Africa, are essential for capturing regional variations and providing a comprehensive understanding of pulmonary hypertension epidemiology [15,16].

## 4. Pulmonary Arterial Hypertension: A Global Health Challenge

The current classification has assigned the majority of infectious diseases to Group 1 (i.e., pulmonary arterial hypertension) [3,4]. This group has diverse pathological and clinical manifestations, making it a complex and heterogeneous condition. In clinical practice, pulmonary arterial hypertension is often considered a single entity, despite its variability, primarily because it groups various clinical entities for targeted therapies in clinical trials. Pulmonary arterial hypertension is a significant global health challenge that has significant regional variations in prevalence and incidence [13,14,17,18]. While recent studies have provided valuable insights, limitations in data collection and the evolving nature of disease definitions underscore the need for ongoing research. Addressing the global impact of pulmonary arterial hypertension requires a focus on regional aspects, particularly in underrepresented areas, and a deeper understanding of the interplay between infectious diseases and pulmonary vascular disorders.

Epidemiological research on pulmonary arterial hypertension has revealed significant regional variations. The initial estimates suggested a prevalence of 2 to 10 cases per million adults annually, but recent studies indicate a wider range of 11 to 60 cases per million adults. The 2021 Global Burden of Diseases, Injuries, and Risk Factors Study (GBD) estimated 192,000 prevalent cases of pulmonary arterial hypertension worldwide, with an age-standardized prevalence rate of 2.28 per 100,000 population and an incidence rate of 0.52 per 100,000. Regional disparities are apparent, with incidence rates ranging from 0.30 per 100,000 in high-income North America to 0.92 per 100,000 in eastern Sub-Saharan Africa. Infectious diseases, genetic predispositions, and environmental factors such as altitude and air quality influence these variations [13,14,18].

## 5. The Role of Helminthic Infection

### 5.1. Schistosomiasis

**Figure 1:**
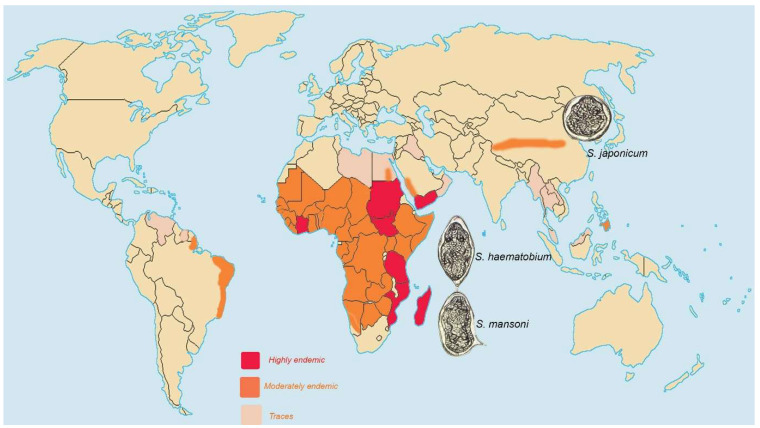
A map illustrating the proportion of the population infected with common human schistosomiasis globally.

Schistosomiasis, one of the most prevalent parasitic diseases globally, affects over 200 million people worldwide and is endemic in 74 countries, including regions in Africa, Brazil, the Middle East, and Southeast Asia [19,20] (Figure 1). The pathological mechanism of schistosomiasis-induced pulmonary hypertension involves an immunological reaction triggered by *Schistosoma* eggs in the lungs, resulting in granuloma formation and subsequent remodeling of pulmonary arterioles [20]. According to estimates, 10% of these patients develop hepatosplenic disease. That may lead to portal hypertension, which opens portosystemic shunts, allowing egg embolization to the lungs, which is thought to be a key factor in the development of pulmonary hypertension [21,22,23,24]. Patients with pulmonary hypertension due to schistosomiasis may present clinical, laboratory, and hemodynamic profiles similar to those observed in pulmonary arterial hypertension [25].

Many studies have shown that around 5 to 30% of individuals with hepatosplenic schistosomiasis develop pulmonary arterial hypertension [22,26]. This suggests that approximately 4 to 10 million people globally may have pulmonary hypertension associated with schistosomiasis [15,27]. Our experiments have demonstrated that Schistosoma infection frequently results in alterations to the pulmonary vasculature. Despite the presence of pulmonary vascular pathology in 46% of infected experimental animals, only 12% displayed signs of right ventricular hypertrophy, which suggests significant pulmonary hypertension. The expectation is that subclinical pulmonary vascular pathology has a high prevalence, but only a small percentage of these cases will be diagnosed with clinical pulmonary hypertension [28]. This suggests that even in asymptomatic individuals, there is a risk of developing pulmonary vasculopathy associated with schistosomiasis.

Significant regional variation is mainly due to the endemicity of various *Schistosoma* species that cause human schistosomiasis. In Africa, schistosomiasis is highly endemic in many parts of Sub-Saharan Africa, with an estimated 90% of the global disease burden occurring in this region. Although Sub-Saharan Africa constitutes about 13% of the world’s population, it accounts for up to 90% of schistosomiasis cases [29,30]. While exact prevalence data for schistosomiasis-associated pulmonary arterial hypertension in Africa is limited, some studies suggest pulmonary hypertension may affect up to 10% of individuals with hepatosplenic schistosomiasis [31]. This indicates that schistosomiasis-associated pulmonary hypertension may affect millions of people in Africa [26]. Chronic infection with *Schistosoma* parasites is the most significant risk factor, particularly *Schistosoma mansoni* [26]. While these are the primary risk factors identified, it is important to note that the precise prevalence and risk factors for schistosomiasis pulmonary arterial hypertension in Africa are not fully understood due to limited diagnostic capabilities and research in many endemic areas. However, co-infection, particularly with HIV, has also been considered an attributed factor in Africa [21,32,33,34].

Schistosomiasis was introduced to South America because of the African slave trade. According to estimates, approximately 1.8 million people in the region, mainly in Brazil, are believed to be infected. At the same time, 25 million are at risk of contracting the disease, primarily because of skin contact with contaminated water [35]. In the Americas, Brazil has the highest burden of schistosomiasis pulmonary arterial hypertension. According to Brazilian studies, 7.7–10.7% of patients with hepatosplenic schistosomiasis develop pulmonary hypertension [36]. In specialized pulmonary hypertension centers in Brazil, such as Belo Horizonte and Salvador Bahia, 20–30% of cases are attributed to schistosomiasis [36,37,38,39,40,41]. Venezuela has been reported to have some cases of schistosomiasis, but specific schistosomiasis pulmonary arterial hypertension prevalence data are limited [42].

Egypt, part of the Nile Delta region, has historically been an endemic area for schistosomiasis. Schistosomiasis has been a significant health problem in Egypt for several decades, primarily due to *Schistosoma haematobium* and/or *Schistosoma mansoni*. Historical data from Egypt reported pulmonary hypertension in 7.7–18.5% of patients with hepatosplenic schistosomiasis [20]. A study conducted in the Nile Delta region of Egypt found that 8.6% of asymptomatic rural residents previously infected with schistosomiasis had pulmonary artery systolic pressure (PASP) > 40 mmHg, indicating pulmonary hypertension [43]. This problem was almost controlled, with the prevalence of *S. haematobium* and *S. mansoni* declining from 15 and 40% in 1982 to 7 and 4% in 2000 [44,45]. However, current prevalence data from this region are limited.

In Asia, schistosomiasis is endemic in parts of China and Southeast Asia, but there is a paucity of data on associated pulmonary hypertension. The prevalence appears lower than in Latin America and Africa, possibly due to differences in *Schistosoma* species and host responses. The most common species causing infection in this region is likely *Schistosoma japonicum*. Schistosomiasis is prevalent in pockets near the Yangtze River in China and certain Southeast Asian regions [46,47]. Recent estimates suggest a prevalence of 4.2% in Hunan province and up to 20% in the Dongting Lake area [48]. A retrospective study from China found that only 10 out of 18,829 (0.053%) people with schistosomiasis had pulmonary hypertension diagnosed by echocardiography [41]. Some reports have claimed *that Schistosoma japonicum* appears to cause less severe pulmonary hypertension than *Schistosoma mansoni* [49]. The prevalence of schistosomiasis in China has decreased significantly over the past decades due to control efforts [50].

In summary, schistosomiasis-associated pulmonary arterial hypertension poses a significant global health challenge, particularly in endemic regions. However, its true prevalence and incidence remain uncertain due to several factors. The lack of systematic screening, limited access to diagnostic tools, and low awareness of this complication in endemic areas contribute to underestimating schistosomiasis pulmonary arterial hypertension prevalence. Variability in diagnostic criteria and the absence of definitive guidelines further complicate accurate assessment. Additionally, differences in prevalence between acute and chronic schistosomiasis and potential geographic variations in host responses and *Schistosoma* species contribute to the complexity of estimating the disease burden. Future research should focus on developing standardized diagnostic criteria, implementing systematic screening programs, improving access to diagnostic tools, and conducting large-scale epidemiological studies. These efforts will enable a more accurate assessment of the global burden of schistosomiasis and pulmonary arterial hypertension and facilitate the development of targeted interventions to mitigate their impact on public health [19,51]. Further details on this condition in Brazil by Louerio et al. [52] are thoroughly discussed in this special issue of Infectious Disease Reports.

### 5.2. Lymphatic filariasis

*Wuchereria bancrofti,* a parasitic filarial nematode known to cause Lymphatic filariasis, has been associated with the development of pulmonary hypertension; the mechanisms linking *W. bancrofti* infection to pulmonary hypertension have not yet been thoroughly investigated. The possible mechanism might be related to intense lung eosinophilic infiltration [53,54]. Furthermore, it has been observed that microfilariae protein appears to be a novel ligand of TLR4 from *W. bancrofti*, inducing immunopathogenesis of filarial infection and resulting in inflammatory consequences. These factors might lead to chronic inflammation, contributing to endothelial damage, fibrosis, and pulmonary vascular remodeling [55,56].

### 5.3. Chinese Liver Fluke

*Clonorchis sinensis*, commonly known as the Chinese liver fluke, is the causative agent of clonorchiasis, a condition associated with liver and biliary tract disorders, including cholangiocarcinoma [57,58]. In rare cases, it can also lead to pulmonary symptoms accompanied by eosinophilia. Hypothetical mechanisms that could explain this link include immunological and chronic inflammation (particularly pulmonary eosinophilia) [59], which may affect the pulmonary vasculature and portal hypertension, contributing to the development of pulmonary hypertension. Further research is necessary to understand this potential association [60]. Although no definitive link has been established, clinicians in endemic regions should remain alert to the possibility of pulmonary complications in patients with *C. sinensis* infection.

### 5.4. Hydatid Diseases

Infections caused by *Echinococcus granulosus* (cystic echinococcosis) or *Echinococcus multilocularis* (alveolar echinococcosis) [61] can lead to pulmonary hypertension through mechanical and immunological mechanisms [62]. Hydatid cysts in the lungs or liver can compress or embolize pulmonary vessels into the pulmonary circulation, causing increased vascular resistance [63,64].

## 6. The Role of Bacterial Infection

### 6.1. Whooping Cough

*Bordetella pertussis*, the causative agent of whooping cough, has been increasingly recognized for its potential to induce severe pulmonary hypertension, particularly in infants, and depends on pertussis toxin expression [65]. The *Bordetella pertussis* toxin is crucial in promoting pulmonary hypertension through a cascade of events triggered by the infection. These include acute pulmonary vasoconstriction and *B. pertussis* T-mediated aggregates of abundant leukocytes in small pulmonary arteries, veins, and lymphatics in fatal cases, which ultimately compromise pulmonary blood flow and exacerbate hypoxemia [66,67]. The link between extreme leucocytosis and pulmonary hypertension in infants with pertussis, particularly during the epidemic peaks in the 1990s in the UK, is critical. Seventy-five percent of infants (under 3 months of age) succumbed to the infection and showed signs of pulmonary hypertension [67,68]. This intractable pathology is a significant risk factor for infection-induced death, often leading to cardiac ischemia, cardiac arrest, and cardiac failure, even when treated with advanced therapies [65].

### 6.2. Tuberculosis

Tuberculosis has emerged as a notable but often overlooked cause of pulmonary hypertension [69,70,71], particularly in regions with endemic tuberculosis. Recent studies have shed light on the prevalence and implications of this association. It is estimated that 9.4% (95% CI 6.3–13.0) develop pulmonary hypertension in active tuberculosis patients. For post-tuberculosis patients, the estimates are equally significant: 67.0% (95% CI 50.8–81.4) in those with chronic respiratory failure, 42.4% (95% CI 31.3–54.0) in hospitalized or symptomatic patients, and 6.3% (95% CI 2.3–11.8) in non-healthcare-seeking outpatients [71]. The association between tuberculosis and pulmonary hypertension has significant implications as it might reduce quality of life and increase mortality [72,73]. In addition, it might cause diagnostic challenges, such as pulmonary hypertension symptoms that may overlap with residual Tuberculosis symptoms, leading to underdiagnosis [74].

In this special issue of Infectious Disease Reports, Jumaar and his colleagues from South [75] highlight that a history of at least one previous episode of tuberculosis and being in a post-TB stage—even after successful treatment—is associated with increased endothelial dysfunction and angiogenesis. These findings suggest a potential predisposition to pulmonary vascular remodeling, which may contribute to the development of pulmonary hypertension.

## 7. The Role of Fungal Infection

Paracoccidioidomycosis, caused by the dimorphic fungus *Paracoccidioides brasiliensis*, is a systemic mycosis endemic to Latin America that can lead to significant pulmonary complications, including pulmonary hypertension [76,77]. The prevalence of pulmonary hypertension in patients with paracoccidioidomycosis is notable, with recent studies showing that approximately 23% of patients with a history of this condition develop late-stage pulmonary hypertension after successful treatment for the infection [78].

The pathogenesis of pulmonary hypertension in paracoccidioidomycosis involves chronic lung inflammation, leading to vascular remodeling and fibrosis. This persistent inflammatory state contributes to an increase in pulmonary vascular resistance. Additionally, significant remodeling of the adventitial layer of small pulmonary vessels, characterized by collagen deposition and myofibroblast activation, has been observed in paracoccidioidomycosis patients [79,80].

## 8. The Role of Viral Infection

### The Human Immunodeficiency Virus (HIV)

**Figure 2:**
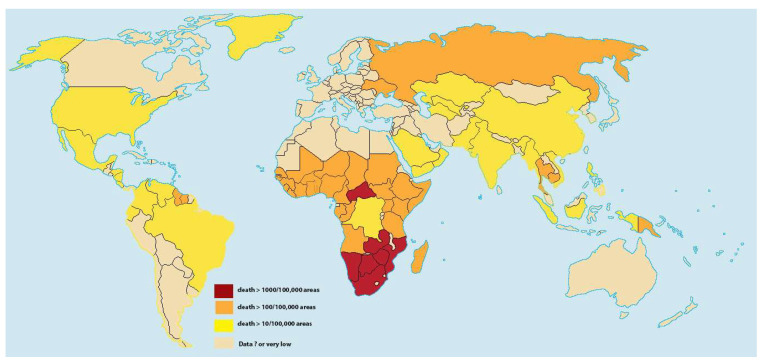
A map illustrating the proportion of the population living with HIV globally.

Pulmonary hypertension has emerged as a significant complication in people living with HIV (Figure 2) [32,81,82,83]. The pathological basis for the link between HIV and pulmonary hypertension remains not fully defined. Still, many recent studies have shed some light on this subject, with limited evidence of the role of HIV viral proteins and systemic inflammation [84,85,86,87]. Further research is needed to understand the cellular metabolism underlying HIV-associated pulmonary arterial hypertension and to increase awareness and early detection of this condition. Subjects with HIV with high viral CD4 count loads (>500 copies/mL) and low CD4 cell counts (<200 cells/μL) were more likely to exhibit elevated pulmonary artery systolic pressure [88,89,90].

The prevalence and incidence of pulmonary hypertension in those with HIV remain significantly higher than in the general population, despite advances in antiretroviral therapy. Combination antiretroviral therapy (ART) has transformed HIV infection from a fatal illness to a chronic disease [91,92]. However, long-term cardiopulmonary complications, including HIV-associated pulmonary arterial hypertension, have become a primary source of morbidity and mortality [93].

A systematic review and meta-analysis conducted in 2019 found that among HIV-infected adults, the prevalence of pulmonary hypertension based on right heart catheterization was 0.5% (95% CI: 0.3–0.60) [94]. However, when using echocardiography as a diagnostic tool, the prevalence rates reported in various studies ranged from 2.6% to 14.0% [95]. A study in France found the prevalence of pulmonary arterial hypertension to be 0.46% in a population of 7648 patients with HIV [96]. This is consistent with earlier estimates from Switzerland, which reported a prevalence of 0.5% in a cohort of 1200 patients with HIV [97]. The prevalence of HIV-associated pulmonary arterial hypertension in a U.S. cohort was reported to be 1.4%, which may reflect the contemporary prevalence in the era of effective antiretroviral therapy [98]. It is also noticed that most participants with HIV-associated pulmonary arterial hypertension came from the southern U.S. census region (61% vs. 36%; *p* = 0.05) [98]. A study by Duncan et al. found a higher incidence of pulmonary hypertension in veterans with HIV (28.6 cases per 1000 person-years) compared to those without HIV (23.4 cases per 1000 person-years) [88].

Regional variations are evident, with higher rates reported in Africa and among specific populations in the United States. The Pan African Pulmonary Hypertension Cohort study found that 36% of HIV-positive patients presented with pulmonary arterial hypertension, which was significantly higher than in HIV-negative individuals (15%) [31]. The interplay of factors such as HIV disease progression, comorbidities, and healthcare access contributes to the complex epidemiology of pulmonary hypertension to HIV. A systematic review and meta-analysis revealed that survival was higher in high-income countries compared with lower-income countries (β 0.50, 95% CI 0.28 to 0.73, *p* < 0.001) and in Europe compared with the Americas (β 0.56, 95% CI 0.37 to 0.75, *p* < 0.001) [91]. This highlights the importance of healthcare access and quality in managing pulmonary hypertension associated with HIV.

Studies using echocardiography tend to report higher prevalence rates than those using right heart catheterization, which is the gold standard for diagnosis. In addition, the disparity in survival outcomes between high-income and low-income countries suggests that access to healthcare and antiretroviral therapy plays a crucial role in the development and progression of pulmonary hypertension related to HIV. Furthermore, the underlying condition may influence the severity and prevalence of pulmonary hypertension related to HIV. Previous tuberculosis infection was significantly more common in HIV-positive patients with pulmonary hypertension compared to HIV-negative patients with pulmonary hypertension (62% versus 18%, *p* < 0.0001) [31,71,99]; co-infection with schistosomiasis can increase the severity of pulmonary hypertension [100]. However, no data on the prevalence of co-infection is available yet. The role of various opportunistic bacterial and parasitic infections in pulmonary hypertension, along with the disparities related to geographic regions and levels of HIV endemicity, remains unclear and requires further investigation. In addition, intravenous drug use has been identified as a significant risk factor, reporting prevalence from 69% to 90% of pulmonary hypertension related to HIV [101,102,103,104].

The importance of screening for pulmonary hypertension is underscored by these findings, especially in resource-limited settings and among high-risk subgroups. Efforts should be made to tackle the disparities in healthcare access and quality between high-income and low-income countries to improve survival outcomes for those who have HIV and pulmonary hypertension.

## 9. The Role of the Microbiome

The human gut microbiome is a vast ecosystem of trillions of microorganisms, including bacteria, archaea, fungi, and viruses. These microbes are crucial in maintaining gut health, digestion, and immune function. Emerging evidence suggests a potential link between dysbiosis (an imbalance in the gut microbiome) and various chronic inflammatory diseases, including pulmonary hypertension [105,106,107,108].

The gut microbiome’s influence on pulmonary hypertension involves intricate mechanisms. Metabolites produced by gut bacteria influence vascular function and inflammation, thereby affecting the development of pulmonary hypertension. Disruptions in the gut microbiome can trigger systemic inflammation, potentially exacerbating pulmonary vascular issues. The gut–lung axis, a bidirectional communication pathway, further contributes to this relationship through microbial translocation, immune cell trafficking, and neuroendocrine signaling. These interconnected processes collectively play a significant role in the complex pathogenesis of pulmonary hypertension [109,110,111,112,113]. Schistosomiasis injection-induced alterations in the gut–lung microbiome can lead to an abnormal phenotype associated with pulmonary arterial hypertension, which has a significant impact on the disease [114]. This is another illustration of how co-infection is a significant factor in the development of pulmonary hypertension generally.

Understanding these mechanisms opens up new possibilities for managing and researching pulmonary hypertension. Potential interventions include microbiome modulation through probiotics or fecal microbiota transplantation, metabolite-based therapies targeting specific microbial products, and the development of microbiome-based biomarkers for assessing pulmonary hypertension risk and monitoring treatment response. As research in this field progresses, it may lead to innovative, personalized approaches to pulmonary hypertension diagnosis and therapy [115].

Prisco et al. [116] presented further details regarding this condition in this special issue of Infectious Disease Reports. Their review examined emerging preclinical and clinical evidence suggesting that alterations in the gut microbiome may either initiate or facilitate the progression of established pulmonary arterial hypertension by modulating systemic immune responses and the contributions of infections to the pathogenesis of pulmonary hypertension.

## 10. Conclusions and Call to Action

The link between parasitic infections and pulmonary hypertension is significant, especially in regions with endemic infections. Clinicians should maintain a high index of suspicion for pulmonary hypertension in patients with a history of parasitic diseases, especially schistosomiasis, which is recognized as a leading global cause of pulmonary hypertension. A multifaceted approach is necessary to address this relationship, encompassing basic scientific research, clinical studies, and public health initiatives. By understanding the mechanisms linking parasitic infections to pulmonary hypertension, we can enhance prevention, early detection, and treatment strategies, ultimately reducing the global burden of infection-related pulmonary hypertension.

Future research should focus on these critical areas to enhance the field through the following steps:

1. Investigate how parasitic infections lead to pulmonary hypertension, with a focus on the role of inflammation in pulmonary vascular remodeling.
2. Conduct extensive studies in endemic regions to better understand the prevalence and risk factors for pulmonary hypertension in populations affected by parasitic infections.
3. Explore how co-infection (polyparasitism) and opportunistic parasitic infections influence the development and progression of pulmonary vascular pathology.
4. Develop and validate novel diagnostic methods, such as biomarkers or advanced imaging techniques, to enable the timely identification of pulmonary hypertension in patients with parasitic infections.
5. Investigate targeted treatments that address infectious agents and the associated pulmonary vascular damage. This includes evaluating antiparasitic therapies alongside interventions aimed at mitigating vascular remodeling.

Our ability to combat parasitic infections and their severe complications, like pulmonary hypertension, can be enhanced by addressing these research priorities, particularly in endemic areas.

## Figures and Tables

**Table 1:** Updated clinical classification of pulmonary hypertension as per the Seventh World Symposium on Pulmonary Hypertension, Barcelona, Spain 2024 [6].

**Group 1: Pulmonary Arterial Hypertension**
1.1 Idiopathic
1.1.1 Long-term responders to calcium channel blockers
1.2 Heritable
1.3 Associated with drugs and toxins
Note: patients with heritable pulmonary arterial hypertension or pulmonary arterial hypertension associated with drugs and toxins might be long-term responders to calcium channel blockers.
1.4 Associated with:
1.4.1 Connective Tissue Disease
1.4.2 HIV infection
1.4.3 Portal Hypertension
1.4.4 Congenital Heart Disease
1.4.5 Schistosomiasis
1.5 Pulmonary Arterial Hypertension with features of venous/capillary involvement
(Pulmonary veno-occlusive disease or pulmonary capillary haemangiomatosis)
1.6 Persistent PH of the newborn
**Group 2: PH associated with left heart disease**
2.1 Heart failure:
2.1.1 With preserved ejection fraction
2.1.2 With reduced or mildly reduced ejection fraction
2.1.3 Cardiomyopathies with specific aetiologies
(Hypertrophic, Amyloid, Fabry disease, and Chagas disease)
2.2 Valvular heart disease:
2.2.1 Aortic valve disease
2.2.2 Mitral valve disease
2.2.3 Mixed valvular disease
2.3 Congenital/acquired cardiovascular conditions leading to post-capillary pulmonary hypertension group
**Group 3: PH associated with lung diseases and/or hypoxia**
3.1 COPD and/or emphysema
3.2 Interstitial lung disease
3.3 Combined pulmonary fibrosis and emphysema
3.4 Other parenchymal lung diseases
(Parenchymal lung diseases not included in the group)
3.5 Nonparenchymal restrictive diseases:
3.5.1 Hypoventilation syndromes
3.5.2 Pneumonectomy
3.6 Hypoxia without lung disease (e.g., high altitude)
3.7 Developmental lung diseases
**Group 4: PH associated with pulmonary artery obstructions**
4.1 Chronic thromboembolic pulmonary hypertension
4.2 Other pulmonary artery obstructions
(Other causes of pulmonary artery obstructions include sarcomas (high- or intermediate-grade or angiosarcoma), other malignant tumors (e.g., renal carcinoma, uterine carcinoma, germ-cell tumors of the testis), nonmalignant tumours (e.g., uterine leiomyoma), arteritis without connective tissue disease, congenital pulmonary arterial stenoses and hydatidosis)
**Group 5: PH with unclear and/or multifactorial mechanisms**
5.1 Hematological disorders
(including inherited and acquired chronic haemolytic anemia and chronic myeloproliferative disorders)
5.2 Systemic disorders
(Sarcoidosis, pulmonary Langerhans cell histiocytosis, and neurofibromatosis type 1)
5.3 Metabolic disorders
(Including glycogen storage diseases and Gaucher disease.)
5.4 Chronic renal failure with or without haemodialysis
5.5 Pulmonary tumor thrombotic microangiopathy
5.6 Fibrosing mediastinitis
5.7 Complex congenital heart disease

## Data Availability

No new data were created or analyzed in this study. Data sharing is not applicable to this article.
